# Evaluating Cortical Alterations in Patients With Chronic Back Pain Using Neuroimaging Techniques: Recent Advances and Perspectives

**DOI:** 10.3389/fpsyg.2019.02527

**Published:** 2019-11-14

**Authors:** Li Zhang, Lili Zhou, Qiaoyue Ren, Tahmineh Mokhtari, Li Wan, Xiaolin Zhou, Li Hu

**Affiliations:** ^1^School of Psychological and Cognitive Sciences, Peking University, Beijing, China; ^2^Beijing Key Laboratory of Behavior and Mental Health, Peking University, Beijing, China; ^3^CAS Key Laboratory of Mental Health, Institute of Psychology, Beijing, China; ^4^Department of Psychology, University of Chinese Academy of Sciences, Beijing, China; ^5^Department of Pain Management, The State Key Clinical Specialty in Pain Medicine, The Second Affiliated Hospital of Guangzhou Medical University, Guangzhou, China; ^6^PKU-IDG/McGovern Institute for Brain Research, Peking University, Beijing, China

**Keywords:** chronic back pain, cortical reorganization, neuroimaging techniques, central sensitization, emotional and cognitive disorders

## Abstract

Chronic back pain (CBP) is a leading cause of disability and results in considerable socio-economic burdens worldwide. Although CBP patients are commonly diagnosed and treated with a focus on the “end organ dysfunction” (i.e., peripheral nerve injuries or diseases), the evaluation of CBP remains flawed and problematic with great challenges. Given that the peripheral nerve injuries or diseases are insufficient to define the etiology of CBP in some cases, the evaluation of alterations in the central nervous system becomes particularly necessary and important. With the development of advanced neuroimaging techniques, extensive studies have been carried out to identify the cortical abnormalities in CBP patients. Here, we provide a comprehensive overview on a series of novel findings from these neuroimaging studies to improve our understanding of the cortical abnormalities originated in the disease. First, CBP patients normally exhibit central sensitization to external painful stimuli, which is indexed by increased pain sensitivity and brain activations in pain-related brain regions. Second, long-term suffering from chronic pain leads to emotional disorders, cognitive impairments, and the abnormalities of the relevant brain networks among CBP patients. Third, CBP is associated with massive cortical reorganization, including structural, functional, and metabolic brain changes. Overall, a deep insight into the neural mechanisms underlying the development and outcome of CBP through more sophisticated neuroimaging investigations could not only improve our current understanding of the etiology of CBP but also facilitate the diagnosis and treatment of CBP based on precision medicine.

## Introduction

As a substantial worldwide health problem, chronic back pain (CBP) is one of the most frequent complaints and the second most common symptom reported by patients during their primary physical care visits (Mantyselka et al., 2001; Vogt et al., 2005). Being more prevalent in females (Hoy et al., 2012; Maher et al., 2017), CBP has been introduced as the first leading cause of years of lived with disability (YLDs) in 2016, with the incidence of 57.6 million YLDs all over the world (Vos et al., 2017). In western countries, the prevalence of lifetime CBP ranges from 49% to 70% (Koes et al., 2006), seriously impairing the quality of life in these patients (Ricci et al., 2006). In addition, CBP imposes considerable socio-economic burdens and leads to rigorous challenges for healthcare program developments (Vogt et al., 2005; Dagenais et al., 2008).

It is widely accepted that CBP can be caused by anatomical abnormalities or systemic diseases in/around the spinal cord (i.e., peripheral level), including lesions or degenerations in certain structures of the spine (Deyo et al., 1992; Dixit and Dickson, 2018). Therefore, in the diagnosis and treatment of CBP, it is reasonable that clinicians commonly focus on the “end organ dysfunction,” where structural and functional abnormalities could be found within the musculoskeletal system (Robinson and Apkarian, 2009; Wand et al., 2011). However, a specific pathoanatomical diagnosis of the pain generators cannot be precisely identified in 90% of CBP patients with apparent symptoms (Koes et al., 2006; Maher et al., 2017), i.e., most CBP patients are non-specific, and characterized by a range of biophysical, psychological, and social factors with an extreme variability in genesis (Hartvigsen et al., 2018). Therefore, it remains difficult to accurately diagnose and evaluate non-specific CBP to date. Consequently, there is no doubt that such imprecise diagnosis and evaluation of CBP hamper the individualized intervention based on the etiology of the disease itself (Wand and O’Connell, 2008; Peng et al., 2017), leading to the prolongation of treatment duration and the deterioration of the health condition in patients (Heithoff and Burton, 1985; Chou et al., 2007).

Theoretically, the imprecise diagnosis of CBP could be caused by the possible dissociation between nociception (nociceptive inputs caused by injuries or diseases at the peripheral level) and pain (a conscious experience in the brain) (Craufurd et al., 1990; Loeser, 1991; Mee et al., 2006; Hu and Iannetti, 2019). Pain can occur in the absence of nociception, and the link between nociception and pain is heavily dependent on various factors, including cognitive condition (e.g., attention, expectation, and context) and emotional state/trait (e.g., depression, anxiety, and catastrophizing) (Loeser, 1991; Rhudy et al., 2006). Based on these theoretical perspectives, accumulating evidence has revealed potential obstacles in diagnosis and treatment of CBP, spanning from the aspects of structural (Apkarian et al., 2004; Schmidt-Wilcke et al., 2006; Baliki et al., 2011b; Seminowicz et al., 2011, 2013; Ivo et al., 2013; Fritz et al., 2016), functional (Giesecke et al., 2004; Baliki et al., 2011a, 2014; Seminowicz et al., 2011; Berger et al., 2014; Mao et al., 2014; Yu et al., 2014; Pijnenburg et al., 2015; Hotz-Boendermaker et al., 2016; Letzen and Robinson, 2017), to metabolic abnormalities in the brain (Grachev et al., 2000, 2001, 2002, 2003; Gussew et al., 2011). Since the properties of the peripheral injury or disease are insufficient to characterize CBP, it would be important to evaluate the cortical abnormalities for a better understanding of the causes and consequences of CBP (Wand et al., 2011; Ng et al., 2018).

In clinical practice, an integrated diagnosis strategy that not only assesses injuries or diseases in/around the spinal cord but also evaluates cortical abnormalities is highly needed to optimize the treatment strategies for CBP patients, especially for non-specific CBP patients. In the present study, we overviewed findings in recent research that evaluates cortical abnormalities in CBP patients using advanced neuroimaging techniques, and discussed some perspectives on how to improve the diagnosis of the disease (please note that there are not enough studies exploring cortical abnormalities in specific CBP patients, and the possible differences of brain alterations in specific and non-specific CBP patients are not considered in the present study).

## Cortical Evaluations Using Neuroimaging Techniques

Several non-invasive neuroimaging techniques with different underlying physical principles are widely adopted to evaluate the cortical abnormalities in CBP patients (Aine, 1995; Chen, 2001), including structural and functional magnetic resonance imaging (MRI; Table 1), electroencephalography (EEG; Table 2), magnetoencephalography (MEG; Table 2), and magnetic resonance spectroscopy (MRS; Table 3). In the following sections, we provided a comprehensive review of recent studies that investigated brain alterations in CBP patients by these techniques.

**TABLE 1:** Evaluation of cortical alterations in CBP patients using structural and functional MRI techniques.

**Author, year**	**Scan type**	**Patients**	**Controls**	**Stimulation**	**Targets**	**Main findings (patients compared with controls)**
Apkarian et al., 2004	Structural MRI	26 CBP (in two subgroups of neuropathic and non-neuropathic pain)	26 Healthy controls	Nil	Neocortical GM volume, Regional GM density	• 5–11% less neocortical GM volume in CBP patients associated with pain duration • Reduced GM density in bilateral dlPFC and right thalamus, correlated with pain features in neuropathic and non-neuropathic conditions
Baliki et al., 2011b	Structural MRI	36 CBP, 28 CRPS, and 20 knee OA	46 Healthy controls	Nil	Total GM volume, regional GM density	• Altered total GM volume only in CBP patients • Specific patterns of reduced GM density for each chronic pain condition based on voxel-wise and gross regional analyses • Significant decrease in GM density of some regions, such as the primary sensory, motor regions, hippocampus, visual cortex, and bilateral INS cortex after longer pain duration • Relating GM density reductions to chronicity of pain
Fritz et al., 2016	Structural MRI	111 CBP	432 Healthy controls	Nil	Regional GM density	• Decreased GM in the vlPFC, dlPFC, vmPFC, dmPFC, and anterior INS in patients • A weak negative correlation between pain severity and GM volume in the left dlPFC, vlPFC, and ACC
Ivo et al., 2013	Structural MRI	14 CLBP	14 Healthy controls	Nil	Total GM volume, total WM volume, and regional GM density	• Decreased total GM volume • Decreased total WM volume • Decreased GM density in areas associated with pain processing and modulation such as dlPFC, thalamus, and MCC
Schmidt-Wilcke et al., 2006	Structural MRI	18 CBP	18 healthy controls	Nil	Regional GM density	• Decreased GM in the brainstem and the somatosensory cortex • A negative correlation between pain intensity and decreased GM in these brain areas • Increased GM in the basal ganglia bilaterally and the left thalamus
Seminowicz et al., 2011	Structural MRI and task fMRI	18 CLBP and 14 CLBP six months after treatment	16 Healthy controls (10 controls revisited)	MSIT	Total GM volume, total WM volume, and partial volume estimation	• Thinner brain cortex in the left dlPFC before treatment • Increased cortical thickness in the left dlPFC after treatment, correlated with the reduction in both pain and physical disability • Increased thickness in the primary motor cortex, correlated with reduced physical disability • Increased thickness in the right anterior INS, correlated with reduced pain • Abnormal left dlPFC activity in task-fMRI evaluations before treatment • Normalized left dlPFC activity in task-fMRI evaluations after treatment
Baliki et al., 2011a	Resting state fMRI	15 CBP	15 Healthy controls	Nil	BOLD fluctuations across different frequencies in different regions of the brain	• Strong low frequency power in the lateral parietal regions, mPFC, PCC, and visual regions • Middle frequency power in middle portions of the ACC, bilateral INS, and subcortical nuclei, including the basal ganglia and thalamus • High frequency power located in the ACC, INS, subcortical regions, temporal poles, and hippocampal formation • A correlation between mPFC aberrant BOLD high frequency dynamics and changed functional connectivity to pain signaling/modulating brain regions
Baliki et al., 2014	Resting state fMRI	18 CBP, 19 CRPS, and 14 knee OA	36 Healthy controls	Nil	ROI and BOLD analysis	• Decreased connectivity of the mPFC to the posterior constituents of the DMN in all patients • Increased connectivity to the INS cortex in proportion to the intensity of pain in all patients • Increased high frequency oscillations in multiple DMN regions, such as the mPFC • Correlation between both phase and frequency alterations and pain duration in OA and CBP patients
Letzen and Robinson, 2017	Resting state fMRI	17 CLBP	16 Healthy controls	Nil	Positive and negative moods altered DMN fMRI patterns	• Significant sadness > baseline interaction in clusters spanning the parietal operculum/postcentral gyrus, INS cortices, ACC, frontal pole, and a portion of the cerebellum • Significant happiness > baseline only in cluster covering a portion of the cerebellum
Pijnenburg et al., 2015	Resting state fMRI and Task fMRI	17 NSLBP	17 Healthy controls	Nil	Sensorimotor functional connectivity and STSTS performance	• Increased time to perform the STSTS task in patients • Decreased resting-state functional connectivity of brain regions associated with sensory and/or motor information integration such as lobule IV and V of the left cerebellum and left precentral gyrus in patients • Decreased functional connectivity correlated with a longer duration of the STSTS task in both NSLBP patients and healthy subjects
Yu et al., 2014	Resting state fMRI	18 CLBP	18 Healthy controls	Nil	Brain resting state PAG-FC alterations	• Increased FC between the PAG and vmPFC/rACC • Negative correlations between pain scores and FC in PAG-vmPFC/rACC after pain-induction maneuver in patients • Negative correlations between CLBP duration and PAG-INS and PAG-amygdala FC before pain-induction maneuver in the patient
Berger et al., 2014	Resting state fMRI and Task fMRI	22 CBP	21 Healthy controls	Monetary decision-making task	Evaluation of modular connectivity of each subjects’ NAc	• Significantly higher sensitivity in CBP patients • Correlation between sensitivity and connectivity within NAc module (with strong connections to the frontal cortex) described by healthy controls • No correlation between sensitivity and connectivity within NAc module (strong connections to subcortical areas) described by CBP patients • High similarity in connectivity between CBP patients and this study’s highly impulsive healthy subjects • Strong correlation between the brain systems that support chronic pain and reward processing • Prediction of the range of behaviors (from simple to complex) from brain activity during rest based on the precedence
Giesecke et al., 2004	Task fMRI	11 Idiopathic CLBP, 16 fibromyalgia	11 Healthy controls	Pressure at neutral site	Sensory testing and regional activation of cortex	• Hyperalgesia in CLBP and fibromyalgia groups • Slightly higher intense pain in the controls than in the CLBP patients with or the with fibromyalgia patients • Applying equal amounts of pressure results in five common detected regions of neuronal activation in pain-related cortical areas including the contralateral S1 and S2, inferior parietal lobule, ipsilateral S2, and cerebellum in CLBP and fibromyalgia patients • Common neuronal activations in three groups when exposing the subjects to the stimuli which evoked subjectively similar pain
Hotz-Boendermaker et al., 2016	Task fMRI	13 CLBP	13 Healthy controls	Non-painful posterior–anterior movement pressure	Reorganization in the S1 and S2 cortices following mechanosensory stimuli	• No cortical reorganization in S1 after stimulation • Reduced activation of S2 in both hemispheres in CLBP patients • Observed a blurring of the somatotopic representation of the lumbar spine in S2 in CLBP patients
Mao et al., 2014	Task fMRI	36 CLBP	36 Healthy controls	MSIT	Cingulo-frontal-parietal cognitive/attention network	• Less activation in the CFP network including the dlPFC, dorsal ACC, and bilateral SPC in attention-demanding task • Low response accuracy in interference trials • A significant negative correlation between the VAS score of pain and activation of the right PFC during performing the MSIT in CLBP patients

*MRI, magnetic resonance imaging; fMRI, functional magnetic resonance imaging; CBP, chronic back pain; CLBP, chronic low back pain; NSLBP, non-specific low back pain; CRPS, complex regional pain syndrome; OA, osteoarthritis; GM, gray matter; WM, white matter; ROI, region of interest; FC, functional connectivity; VAS, visual analog scale; MSIT, multi-source interference task; DMN, default-mode network; STSTS, sit-to-stand-to-sit task; NAc, nucleus accumbens; rCBF, regional cerebral blood flow; PFC, prefrontal cortex; dlPFC, dorsolateral prefrontal cortex; vlPFC, ventrolateral prefrontal cortex; vmPFC, ventral medial prefrontal cortex; dmPFC, dorsal medial prefrontal cortex; mPFC, medial prefrontal cortex; MCC, middle cingulate cortex; PCC, posterior cingulate cortex; LPC, lateral parietal cortex; DMN, default-mode network; INS, insula; PAG, periaqueductal gray; rACC, rostral anterior cingulate cortex; S1, primary sensory cortex; S2, secondary sensory cortex; SPC, superior parietal cortex.*

**TABLE 2:** Evaluation of cortical alterations in CBP patients using EEG and MEG techniques.

**Author, year**	**Scan type**	**Patients**	**Controls**	**Stimulation**	**Main findings**
					**(patients compared with controls)**
Diers et al., 2007	EEG	14 CLBP	13 Healthy controls	Electrical stimuli	• Larger N80 component after stimulation • No significant group difference in the N150 component • Smaller P260 component after stimulation • Positive correlation between N80/N150 amplitudes and perceptual sensitization • Increased perceptual sensitization and increased processing of the sensory-discriminative aspect (N80 component) of pain in patients
Flor et al., 2004	EEG	16 CBP, 16 THA	16 Healthy controls	Electrical stimuli	• Significantly lower pain threshold and pain tolerance in CBP patients compared with THA patients and healthy controls • Reduced habituation in CBP patients • No significant differences in amplitudes of N150, P260, P300, and N500 among three groups • Lower stimulation intensity in CBP patients
Flor et al., 1997a	EEG and MEG	10 CBP	Nine healthy controls	Standard intracutaneous electrical stimuli to the left back and index finger with a non-painful and a painful intensity	• Enhanced power of the evoked early magnetic field (< 100 ms) in LBP patients than healthy controls following painful back stimulation • Medial shift in the maximum activity elicited in the S1 in LBP patients
Flor et al., 1997b	EEG	12 CBP	12 Healthy controls	Pain- and body-related verbal materials	• No more recognition of patients in the pain-related words • Enhanced N100 and N200 of the left hemisphere to pain-related words, when compared to neutral words • A positive shift to all words extending into the 800 ms range • No distinct P300 in CBP patients • Enhanced levels of skin conductance to the pain-related words
Tamburin et al., 2014	EEG	12 CLBP	12 Healthy controls	IGT	• Lower scores of cognitive measures (MCST) in CLBP patients influenced by pain intensity and duration • Worse performance and the absence of a learning process during the behavioral IGT test with no effect of pain features in CLBP patients • Poor performance in the MCST and the IGT in CLBP patients • The FRN amplitude in wins was higher than in losses in controls, while the opposite happened in CLBP patients • The P300 amplitude was higher in wins than in losses in controls, while there was no difference in CLBP patients
Wiech et al., 2000	EEG	10 CBP	Nine healthy controls	Electrical stimulation	• Somatotopic organization of the S1 • Correlation between the amount of reorganization and pain rating

*EEG, electroencephalography; MEG, magnetoencephalography electromyographic activity; ms, millisecond; CBP, chronic back pain; CLBP, chronic low back pain; THA, tension headache; MCST, modified card sorting test; IGT, Iowa gambling task; ERPs, event-related potentials.*

**TABLE 3:** Evaluation of cortical alterations in CBP patients using the MRS technique.

**Author, year**	**Methods**	**Patients**	**Controls**	**Main findings (patients compared with controls)**
Grachev et al., 2000	^1^H-MRS	Nine CLBP	11 Healthy controls	• Alterations in the human brain chemistry in patients • Decreased NAA and Glu in the dlPFC • No chemical concentration differences in brain regions, such as the cingulate, sensorimotor, etc. • Abnormal interrelationship between chemicals within and across brain regions • A specific correlation between regional chemical concentration and perceptual scores of anxiety and pain
Grachev et al., 2001	^1^H-MRS	Nine CBP	16 Healthy controls	• Alterations in NAA levels of the dlPFC and OFC • Correlations between the levels of brain regional NAA (the OFC and dlPFC) and perceptual measures of pain in CBP patients • Correlation between the NAA changes of the OFC and measures of anxiety in CBP patients
Grachev et al., 2002	^1^H-MRS	12 CLBP with symptoms of anxiety	16 Healthy controls	• An exact correlation between perception and brain chemical contents • The dlPFC and OFC were considered as the best related chemical-perceptual network to pain • The relationship between chemical-anxiety networks was best related to the OFC chemistry in controls and to the dlPFC, OFC, cingulate, and thalamus in CLBP patients • The region best related to the affective component of pain was the cingulate cortex
Grachev et al., 2003	^1^H-MRS	10 CBP with depression	10 Healthy controls	• Decreased NAA levels in the right dlPFC • Strong correlation between depression levels of CBP patients and the levels of NAA levels in the right dlPFC • Weak correlation between the levels of pain levels and levels of NAA in the right dlPFC of CBP patients (compared to depression-NAA correlations)
Gussew et al., 2011	^1^H-MRS	10 CLBP	10 Healthy controls	• Decreased levels of Glu in the ACC • Decreased levels of Gln in the anterior INS, ACC, and thalamus • Decreased levels of NAA in the anterior INS and ACC • Decreased levels of mI was reduced in the ACC and thalamus • No significant changes for Cr
Sharma et al., 2011	^1^H-MRS	11 CLBP	11 Healthy controls	• Correlations between metabolite concentrations and pain characteristics • Decreased NAA and Cho in the left S1 • Lower correlations between all metabolites (NAA, Cho, mI, Glu, and Gln) in the right S1 • Higher and significant correlations between left and right mI levels and between left mI and right Cho • Negative correlation between left and right NAA levels and pain duration • Positive correlation between right Glu/Gln concentrations and pain severity • Significant changes in the neuronal–glial interactions in S1
Sharma et al., 2012	^1^H-MRS	19 CLBP	14 Healthy controls	• Lower right M1 NAA • No significant differences in the Left M1 NAA and mI • No significant correlations between pain characteristics and M1 neurochemical contents
Siddall et al., 2006	^1^H-MRS	32 CLBP	33 Healthy controls	• Significant differences in the chemical levels of ACC, thalamus, and PFC of patients compared with the ones of healthy subjects with accuracies of 100%, 99%, and 97%, respectively

*^1^H-MRS, single-voxel proton magnetic resonance spectroscopy; CBP, chronic back pain; CLBP, chronic low back pain; NAA, *N*-acetyl aspartate; Glu, glutamate; Gln, glutamine; Cr, creatine; mI, myo-inositol; Cho, choline; PFC, prefrontal cortex; dlPFC, dorsolateral prefrontal cortex; OFC, orbital frontal cortex; INS, insula; S1, primary somatosensory cortex; M1, primary motor cortex.*

### Structural and Functional MRI Studies

The structural MRI could provide anatomical information of the brain with high spatial resolution, and the functional MRI (fMRI) is used to determine the location of the “activate” brain regions during cognitive tasks (Aine, 1995; Lindquist, 2008; Huettel et al., 2009; Sadek, 2012). Both techniques are helpful to provide information about brain organization and offer potential new criteria for assessing the neurological status and neurosurgical risk; thus they are widely employed to characterize structural or functional brain alterations among CBP patients under clinical settings (Wand et al., 2011; Ng et al., 2018). Since these neuroimaging techniques have distinct advantages, we reviewed studies that explore the cortical abnormalities in CBP patients using structural MRI, resting state fMRI, and task fMRI, respectively.

By extracting morphological features from structural MRI through some advanced analysis techniques (e.g., voxel-based morphometry), several crucial anatomical changes have been observed in CBP patients. In a pilot study, CBP patients exhibited decreased neocortical gray matter (GM) volume (5–11% less than healthy controls), with the magnitude equivalent to the loss quantity caused by 10–20 years of normal aging (Apkarian et al., 2004). Meanwhile, several studies have reported that the GM density of CBP patients was significantly reduced in a series of pain-related brain regions, including the dorsolateral prefrontal cortex (dlPFC) (Apkarian et al., 2004; Schmidt-Wilcke et al., 2006; Fritz et al., 2016), dorsomedial prefrontal cortex (dmPFC) (Fritz et al., 2016), ventrolateral prefrontal cortex (vlPFC) (Fritz et al., 2016), ventromedial prefrontal cortex (vmPFC) (Fritz et al., 2016), primary somatosensory cortex (S1) (Schmidt-Wilcke et al., 2006; Baliki et al., 2011b; Ivo et al., 2013), secondary somatosensory cortex (S2) (Schmidt-Wilcke et al., 2006; Baliki et al., 2011b; Ivo et al., 2013), insula (INS) (Baliki et al., 2011b; Fritz et al., 2016), middle cingulate cortex (Ivo et al., 2013), thalamus (Apkarian et al., 2004; Ivo et al., 2013), and brainstem (Schmidt-Wilcke et al., 2006). Please note that these brain regions are also functionally associated with some clinical symptoms (e.g., subjective ratings of pain intensity, emotional disorders, and cognitive impairments) of CBP, and the relevant findings are detailed in the following paragraphs. In addition, the GM densities of some brain regions (dorsal rostral pons and somatosensory cortices) demonstrated significant negative correlations with both the subjective ratings of pain intensity and unpleasantness (Schmidt-Wilcke et al., 2006). Importantly, the structural abnormalities in the brain can be reversed by effective CBP treatments. For example, increased cortical thickness in the left dlPFC after treatment could be observed in CBP patients compared to that before treatment, and such improvement in brain structure was positively correlated with the reduction of pain and physical disability (Seminowicz et al., 2011, 2013). All these morphological findings indicated that CBP is accompanied by brain atrophy in regions commonly associated with pain processing and modulation, which has a great influence on pain chronicity (Li and Hu, 2016).

As an effective technique to map white matter (WM) tractography in the brain (Pierpaoli et al., 1996; Basser and Jones, 2002), diffusion tensor imaging (DTI) has been used to study the WM architecture and integrity in CBP patients (Buckalew et al., 2010; Čeko et al., 2015). Lower WM integrity of the splenium of the corpus callosum was found in disabled CBP patients (Buckalew et al., 2010), and importantly, negative correlation between total months of CBP and WM integrity of the splenium of the corpus callosum was observed. In addition, Vania Apkarian and his colleagues tracked brain properties in subacute back pain patients longitudinally for 1 year (Mansour et al., 2013) and 3 years (Vachon-Presseau et al., 2016), as their pain either recovered or transitioned to chronic pain. Testing the role of the corticolimbic system in the development of CBP, Vania Apkarian and his colleagues observed that the dorsal medial prefrontal cortex (mPFC)-amygdala-nucleus accumbens network contributing to risk of chronic pain, which suggested that corticolimbic neuroanatomical factors were important features to predispose subacute back pain patients to recover from or transition to chronic pain (Vachon-Presseau et al., 2016).

The resting state fMRI and task fMRI are normally applied to investigate functional alterations in CBP patients by measuring the spontaneous blood-oxygen-level dependent (BOLD) activities of brain networks at resting state (Baliki et al., 2006; Barkhof et al., 2014; Vachon-Presseau et al., 2019; Zhang et al., 2019) and the evoked BOLD responses during pre-defined tasks/stimuli (Aine, 1995; Lindquist, 2008), respectively. Previous studies using resting state fMRI revealed that CBP patients exhibited reduced deactivation in the mPFC, amygdala, and posterior cingulate cortex, which were considered as key brain regions in the default mode network (DMN) (Baliki et al., 2008). The disruptions of the DMN were related to the cognitive and behavioral impairments associated with chronic pain (Baliki et al., 2008). Additionally, the resting state functional connectivity (FC) of the DMN network was reported to be influenced by negative mood in CBP patients, which implied that the abnormalities of the DMN were related to the information processing of affective-motivational aspect of pain (Letzen and Robinson, 2017). Apart from the DMN network, decreased resting state FC of the sensorimotor network was also observed in CBP patients, which was associated with the performance of a dynamic sensorimotor task (i.e., the duration of performing the sit-to-stand-to-sit task) (Pijnenburg et al., 2015). Notably, increased resting state FC between periaqueductal gray (PAG, a key region in the descending pain modulation pathway) and vmPFC/rostral anterior cingulate cortex (ACC) was represented in CBP patients compared to the one in healthy controls, suggesting an abnormal function of the PAG-centered descending pain modulation system in CBP patients (Yu et al., 2014). Additionally, abnormal FC between mPFC/ACC and brain regions within the DMN was observed in CBP patients, and the abnormal FC was also found to be correlated with pain duration, pain severity, and pain interference (Tu et al., 2019a). Importantly, Vania Apkarian and his colleagues focused on investigating the neural mechanism associated with pain chronification, and obtained several novel findings. For example, they found that corticostriatal FC (between nucleus accumbens and prefrontal cortex) is an accurate predictor of the transition from acute to chronic pain (Baliki et al., 2012; Vachon-Presseau et al., 2016). In addition, they observed that brain activity associated with acute/subacute back pain is limited to regions involved in acute pain, while brain activity related to chronic pain is confined to emotion-related circuitry (Hashmi et al., 2013). This observation suggested that brain representation for back pain can undergo large-scare shifts in brain activity with pain chronification.

Consistently, evidence from the task fMRI revealed that CBP patients exhibited abnormal brain functions related to pain processing (Giesecke et al., 2004). For example, relative to the healthy controls, the CBP patients reported significant higher pain intensity when received painful pressure with fixed physical intensity, and showed stronger activations in several pain-related brain regions, including the contralateral S1, bilateral S2, inferior parietal lobule, and cerebellum (Giesecke et al., 2004), which indicated that CBP patients have increased pain sensitivity. In contrast, when receiving non-painful movement pressure, CBP patients showed a decreased somatosensory acuity and reduced activations of bilateral S2, suggesting a reorganization of higher order processing of sensory information in these patients (Hotz-Boendermaker et al., 2016). In addition, brain dysfunction in emotional and cognitive disorders caused by the maladaptation to chronic pain was frequently reported (Seminowicz et al., 2011; Berger et al., 2014; Mao et al., 2014). For example, in an attention-demanding cognitive task, the impaired cognitive ability and abnormal activation of cingulo-frontal-parietal (CFP) cognitive/attention network (Mao et al., 2014), especially in the dlPFC (Seminowicz et al., 2011), were observed in CBP patients. Further, risky monetary behavior and altered connectivity of the nucleus accumbens (a key brain region in reward processing) were observed in CBP patients (Berger et al., 2014), and such observation has been interpreted as a consequence of cognitive disorders or comorbidity of chronic pain.

### EEG and MEG Studies

Different from MRI techniques that could provide massive spatial information related to cortical regions/networks involved in pain processing, EEG/MEG techniques can measure the cortical changes with a high temporal resolution, thus giving a deep insight into the dynamic process of pain information processing (Chen, 2001; Kucyi and Davis, 2015). Nowadays, crucial progress has been made in the evaluation of cortical dysfunction in CBP patients with EEG/MEG techniques. It is generally accepted that the central sensitization (represented by reduced pain threshold, pain tolerance, and increased perceived pain intensity) and the cortical processing of the sensory-discriminative aspect of pain were significantly enhanced in CBP patients (Flor et al., 1997b, 2004; Diers et al., 2007). For example, a larger amplitude of the early N80 component in somatosensory event-related potentials (ERPs) elicited by painful electrical intramuscular and intracutaneous stimuli was observed in CBP patients (Diers et al., 2007), indicating a central sensitization among these patients. Accordingly, when receiving intracutaneous electrical painful stimuli, CBP patients showed significant larger power of early evoked MEG response than healthy controls did, and the power of this early response was positively correlated with the chronicity in CBP patients (Flor et al., 1997a), which provided a strong evidence that pain chronicity is accompanied with central sensitization, resulting in the abnormal information processing of the sensory-discriminative aspect of pain (Flor et al., 1997a; Diers et al., 2007).

In addition, CBP patients showed evident abnormalities in emotional and cognitive functions. For example, when being assessed the emotional decision-making abilities using the Iowa gambling task, CBP patients scored much lower than healthy controls did, and their performance was significantly influenced by the duration and intensity of their chronic pain (Tamburin et al., 2014). Consistent with this behavioral result, the ERP data showed abnormal feedback processing in CBP patients during the Iowa gambling task (Tamburin et al., 2014). Specifically, the amplitude of feedback-related negativity (FRN) was higher in wins than in losses in healthy controls, while the opposite results were obtained in CBP patients; the amplitude of P300 was higher in wins than in losses in healthy controls, whereas no significant difference was observed in CBP patients. The abnormal feedback cognitive processing resulting in the impairments in the work and family settings were often reported by CBP patients (Tamburin et al., 2014). Moreover, CBP patients showed a lower amplitude of the later P260 component in somatosensory ERPs evoked by painful electrical stimuli, which also suggested the deficiency of higher cognitive functions in CBP patients (e.g., the function related to affective distress) (Diers et al., 2007).

Accompanied by the long-term changes of cortical function, cortical reorganization in CBP patients due to the processes of neuronal plasticity was well documented (Flor et al., 1997a; Wiech et al., 2000). Demonstrated by an MEG study, alterations in the somatotopic organization of the S1 were observed in CBP patients (Wiech et al., 2000). Specifically, being elicited by intracutaneous electrical stimuli with different intensities (from non-painful to painful), the maximal response in the primary somatosensory cortex was shifted more medially in CBP patients than in healthy controls (Flor et al., 1997a). Importantly, such brain reorganization was correlated with subjective pain ratings (Wiech et al., 2000). In summary, chronic pain is accompanied by cortical reorganization, an important neural marker indicating the persistence of the pain experience and the dysfunction of cortical processing. However, the potential relationships between findings obtained using EEG/MEG and MRI techniques in evaluating cortical alterations in CBP patients remain to be elucidated.

### MRS Studies

Chemical changes in the brain of CBP patients can be detected using *in vivo* single-voxel proton MRS (^1^H-MRS), which is able to provide additional evidence on abnormal brain alterations associated with chronic pain (Gussew et al., 2011; Zhao et al., 2017). Several MRS studies showed that a reduced level of *N*-acetyl aspartate (NAA) was observed in several brain regions of CBP patients, including the dlPFC, orbitofrontal cortex (OFC), anterior INS, ACC, and thalamus (Grachev et al., 2000, 2003; Gussew et al., 2011; Sharma et al., 2011, 2012). In addition, some studies reported that CBP patients had reduced levels of glutamate (Glu) in the ACC (Gussew et al., 2011), glucose in the dlPFC (Grachev et al., 2000), and myo-inositol (mI) in the ACC and thalamus (Gussew et al., 2011). These brain chemical imbalances were negatively correlated with pain intensity in CBP patients (Grachev et al., 2000). Importantly, certain changes in brain chemistry were shown to be highly correlated with psychological factors (Siddall et al., 2006). For example, the levels of NAA in the right dlPFC and OFC were, respectively, correlated with depression (Grachev et al., 2003) and anxiety (Grachev et al., 2001, 2002) levels in CBP patients. Therefore, it would be reasonable to hypothesize that brain chemistry changes play an important role in the development and maintenance of CBP and its comorbidity (e.g., depression and anxiety) (Grachev et al., 2000, 2001, 2002, 2003). Given that the reduced Glu level may indicate disordered glutamatergic neurotransmission, and the reduced levels of NAA and mI could be related to the loss of neurons and glial cells (Gussew et al., 2011), these alterations in the brain biochemical profile in CBP patients could represent the cortical reorganization caused by long-term pain suffering (Grachev et al., 2000, 2001, 2002, 2003).

## Discussion

Chronic back pain is a substantial worldwide health problem. The need for treatment of CBP based on a deeper understanding of its causes and outcomes is urgent and pressing in clinic. Considering that chronic pain is associated with several psychological disorders (e.g., depression, anxiety, and sleep disturbances) involving cortical dysfunction, more and more researchers shift their interests from the peripheral level to the cortical level. With the development of non-invasive neuroimaging techniques, a series of novel findings were obtained in this field.

First, despite CBP patients have sensorimotor impairments (e.g., decreased sensitivity to innocuous stimuli), they have enhanced central sensitization to external painful stimuli, manifested by increased subjective pain sensitivity and increased brain activations in pain-related brain regions. It has been reported that increased pain sensitization and decreased sensitivity to innocuous stimuli in CBP patients are associated with increased catastrophizing, which is also linked with increased clinical back pain (Meints et al., 2019). This central sensitization could serve as an overgeneralized protective function to prevent the injured spinal cord from being irritated by the harmful sensory inputs (Apkarian and Reckziegel, 2019). However, since the nociceptive signals induced by external stimuli could be amplified in the ascending pain modulation pathway at any level (e.g., the spinal cord, brainstem, and cerebral cortex), we could not determine the site of central sensitization based on the observation of overactivation in the brain (Apkarian and Reckziegel, 2019). In other words, the perceptual sensitization observed in the human brain could be caused by the changes in sensitivity of the spinal cord, of which the nociceptive information was amplified before propagated to the cortex. Integration of spinal cord and brain MRI/fMRI techniques is warranted to solve this issue (Wand et al., 2011; Ng et al., 2018), which could also allow us to identify relevant neural mechanisms to determine the target site for optimizing the CBP intervention (Wand et al., 2011; Ng et al., 2018).

Second, suffering from chronic pain, CBP patients commonly exhibited emotional and cognitive disorders, including depression, anxiety, catastrophizing, and sleep disturbances (Baliki et al., 2008). In line with these dysregulations, cortical abnormalities have been frequently observed in the regions, pathways, and networks associated with neural processing of emotional and cognitive information in CBP patients (Grachev et al., 2001, 2002, 2003; Baliki et al., 2008; Letzen and Robinson, 2017). In addition to the physiological and psychological factors that are important for the development and maintenance of chronic pain, a biopsychosocial model of pain, which also highlighted the social factors (e.g., interpersonal relationship), has been proposed to better identify the mechanisms of chronic pain (Peng et al., 2017). Importantly, the biopsychosocial model describes pain as a multidimensional and dynamic integration of physiological, psychological, and social factors, which are needed to be considered in the development, maintenance, and treatment of chronic pain (Riedel and Neeck, 2001; Peng et al., 2017). Indeed, more neuroimaging studies under the framework of the biopsychosocial model should be conducted in the future to achieve a comprehensive and sophisticated understanding of the neural mechanisms related to the causes and outcomes of CBP.

Additionally, accumulating evidence has demonstrated that CBP is associated with clear cortical reorganization and neuronal plasticity, which is normally quantified by structural (e.g., GM volume and density) (Apkarian et al., 2004), functional (e.g., cortical representation of the body, brain abnormalities of cortical regions and networks) (Baliki et al., 2008; Hotz-Boendermaker et al., 2016), and metabolic (e.g., levels of NAA, Glu, and mI) (Grachev et al., 2000, 2001, 2002, 2003; Gussew et al., 2011) changes in the brain. Interestingly, the cortical reorganization is reversible by effective treatment (Seminowicz et al., 2011, 2013), suggesting that the quantified brain changes could be used as important neural indicators to monitor the progress of CBP development and to evaluate the effectiveness of CBP treatments, such as acupuncture (Hashmi et al., 2014; Tu et al., 2019b), placebo (Vachon-Presseau et al., 2018), and other pain management approaches (Mülller-Schwefe et al., 2017; Foster et al., 2018).

To sum up, with the development of neuroimaging techniques, great progress has been made to improve our understanding of cortical alterations in CBP patients over the past few years. However, the neural mechanisms associated with the development of CBP remain largely mysterious, which hampers the improvement of the efficacy of CBP treatment. To address this issue, integration of neuroimaging techniques and other biotechnologies (e.g., genetic testing and psychological testing) would be important to achieve a comprehensive assessment of the risk factors (e.g., genetics, injuries, and mental health problems) of the development and maintenance of CBP. In addition, longitudinal studies are highly needed to assess the temporal relationship between chronic pain and neural plasticity. It is worthwhile to note that longitudinal studies would not only improve our understanding of the neural mechanisms associated with the causes and outcomes of CBP but also provide theoretical bases for accurate diagnoses of CBP patients. Integrating the results obtained from comprehensive and longitudinal studies is a promising way to identify the causes of pain and pain-associated comorbidities and deepen our understanding of the mechanisms involved in chronic pain, and ultimately promotes the development of more appropriate and effective treatments in CBP management.

## Author Contributions

LZa, XZ, LW, and LH conceived of this topic. LZa conducted the literature search and wrote the manuscript. LZa, LZo, QR, TM, LW, XZ, and LH revised the work. LZa, XZ, LW, and LH edited the manuscript.

## Conflict of Interest

The authors declare that the research was conducted in the absence of any commercial or financial relationships that could be construed as a potential conflict of interest.
